# The cathartic dream: Using a large language model to study a new type of functional dream in healthy and clinical populations

**DOI:** 10.1111/jsr.70001

**Published:** 2025-02-09

**Authors:** Lampros Perogamvros, Vincent Rochas, Jean‐Baptiste Beau, Virginie Sterpenich, Laurence Bayer

**Affiliations:** ^1^ Center for Sleep Medicine, Department of Psychiatry Geneva University Hospitals Geneva Switzerland; ^2^ Department of Basic Neurosciences University of Geneva Geneva Switzerland; ^3^ M/EEG & Neuromod Platform Fondation Campus Biotech Geneva Geneva Switzerland; ^4^ Oniri Lausanne Switzerland

**Keywords:** artificial intelligence, catharsis, dreams, emotions, large language model

## Abstract

According to some theories of emotion regulation, dreams could modify negative emotions and ultimately reduce their intensity. We introduce here the idea of *cathartic dream*, a specific and separate type of emotional dream, which is characterized by a dynamic plot with emotional twists, and where negative emotions are expressed and ultimately decreased. This process would reflect psychological relief (catharsis according to the Aristotelian definition) and fulfil an emotion regulation function. We developed and validated a tool using a large language model to emotionally categorize the different dreams from dream diaries. Based on this tool, we were able to detect the prevalence of cathartic dreams in datasets of both healthy participants and patients with nightmares. Additionally, we observed the increase of cathartic dreams during 2 weeks of imagery rehearsal therapy and targeted memory reactivation during rapid eye movement sleep. We also demonstrate how the increase of cathartic dreams correlates significantly with the decrease of depression scores in patients with nightmares under therapy, thus supporting their likely functional role in well‐being and their distinct nature among other emotional dreams.

## 1 INTRODUCTION

Dreams are mental experiences that occur during sleep. Even if several hypotheses have associated dreams with memory and emotions, their functions are still unclear. Emotions are a stable feature of dreaming (Windt, 2015), as they are present in many dreams (Merritt, Stickgold, Pace‐Schott, & Williams, 1994; Perogamvros et al., 2017; Sikka et al., 2014). Systematic analyses of dream content indicate that emotions in dreams are varied between positive, negative and neutral ones. The first similar studies proposed a predominance of negative emotions linked to fear (fear, anxiety, apprehension; Hall & Van de Castle, 1966; Merritt, Stickgold, Pace‐Schott, & Williams, 1994; Nielsen et al., 1991), followed by anger and sadness. This finding could be explained by recall bias: negative emotions remain more anchored in memory (Kensinger, 2009) and are more explicitly reported than positive ones in dreams (Schredl & Doll, 1998). Other authors have shown more recently that positive emotions are also very common (Fosse et al., 2001; Malcolm‐Smith et al., 2012; Perogamvros et al., 2017; Sikka et al., 2014). Some dreams (the so‐called “balanced dreams”) can also contain both positive and negative emotions within the scenario (Röver & Schredl, 2017; Sikka et al., 2014), with the presence of emotional shifts from negative to positive emotions and vice‐versa (Masset et al., 2023; Merritt, Stickgold, Pace‐Schott, Williams, & Hobson, 1994). Despite their prevalence at 9%–16% of all dreams (Röver & Schredl, 2017; Sikka et al., 2014), this type of dreams has not been sufficiently studied in the literature.

Several theories support the idea that dreams can act as an emotional “thermostat” (Cartwright et al., 2006; Nielsen & Levin, 2007). More specifically, evidence supports the idea that the expression/resolution of negative emotions in dreams is associated with their reduction during wakefulness. Cartwright (Cartwright, 1991; Cartwright et al., 2006) showed for instance that divorcing participants who incorporate their ex‐partner and overcome the breakup in their dreams are psychologically more adjusted and less depressed than those who do not dream about their divorce or whose dreams about the divorce repeat elements of distress and helplessness. In other words, people who overcome divorce‐related depression are those who dream about the ex‐spouse or elements related to the divorce, and who connect negative affects to other memory elements. Moreover, people experiencing grief have more positive than negative emotions for the deceased in their dreams, which would ultimately contribute to a better coping of grief (Black et al., 2019). These studies support the idea that some dreams may serve a compensatory function (Jung, 1969), in relation to the waking life, by offering possible solutions or suggestions to conflicts (problem‐solving).

Although most of the aforementioned studies have some limitations, as they are correlational and not causal, they strongly suggest that emotionally reworked content during dreams can occasionally lead to the alleviation of emotional conflicts and the resolution of problems during our waking life. The exposure to negative emotions and their transformation to positive ones is the core idea of *catharsis* (κάθαρσις in Greek), as introduced by Aristotle in his work “Poetics”. He says: “*Tragedy is the representation of heroic and complete action: it represents humans in action and, through pity and fear, it provides catharsis [relief] from these and other similar emotions*” (Poetics 1449 b 27–28; Aristotle, 1961). According to the philosopher, theatre and, more specifically, tragedy, can be a form of education that promotes emotional development. For him and other ancient Greeks, experiencing strong negative emotions, such as rage, grief or pity, while watching a tragic play was an emotional and spiritual healing for the individual. Catharsis, as defined by Aristotle, does not just constitute the expression of a negative emotion but, above all, its self‐regulation and inhibition by a new, more positive emotion, such as relief or joy. This transformation is therefore necessary for the phenomenon of catharsis to be effective, as is also the case for extinction, cognitive reappraisal and exposure therapy. Catharsis is therefore a kind of new emotional learning.

We have recently (Schwartz et al., 2022) applied imagery rehearsal therapy (IRT) in patients with nightmare disorder (ND), by asking them to reimagine their nightmares with positive or less negative outcomes. In parallel, in half of these patients, we applied the targeted memory reactivation (TMR) technique (Oudiette & Paller, 2013) during rapid eye movement (REM) sleep to strengthen the positive outcome of IRT. TMR accelerated IRT by significantly decreasing nightmares, while increasing the prevalence of positive emotions, such as joy. In other studies with healthy participants, TMR during REM sleep helped consolidate emotional associative memories and generalization (Sterpenich et al., 2014), while it also increased the positive valence of initially negative stimuli (Hutchison et al., 2021; Rihm & Rasch, 2015). We could then hypothesize that associating a transformed dream scenario (negative to positive) with a sound, as in Schwartz et al. (2022), and replaying this association during REM sleep with TMR over several consecutive nights would promote emotional cathartic processes.

In this study, we aimed to study the emotional dreams in healthy participants and patients with ND, and characterize the prevalence of catharsis. To do so, and partially inspired by previous studies (Schredl & Doll, 1998; Sikka et al., 2014; Zadra & Donderi, 2000), we have first defined five main categories of dreams: neutral, positive, nightmares, bad dreams and cathartic dreams. The last category (cathartic dreams) will be considered as one including dynamic dreams, i.e. emotionally negative dreams that are transformed into a more positive scenario (see also later definition in “Methods”). To score dream content for these emotional categories, we have developed a new artificial intelligence (AI) classification using a large language model (LLM; Cascella et al., 2024). Following the validation of this algorithm against conventional human classification, we were able to use this automatic scoring tool, which offers a distinct advantage in terms of time cost, objectivity and reproducibility for the analysis of large datasets of dreams (Elce et al., 2021). By means of this tool, we have studied the prevalence of the five dream categories in 115 healthy participants and in 36 patients with ND that underwent IRT and TMR (Schwartz et al., 2022). We have hypothesized that, apart from a decrease of negative dreams (nightmares and bad dreams), the number of cathartic dreams will be increased, after successful IRT and TMR during REM sleep, reflecting the positive effect of both these interventions on the content of dreams. Finally, based on similar work from Rosalind Cartwright (Cartwright, 1991; Cartwright et al., 2006), we hypothesized that the cathartic dream will be functionally distinguished by the other types of dreams, by being the only to be correlated to depression scores of patients with ND.

## 2 METHODS

### 2.1 Populations

#### 2.1.1 Healthy participants

One‐hundred and fifteen individuals (75 women, 40 men), aged between 18 and 37 years (M = 22 years, SD = 3.19), were included. Participants had a constant sleep schedule during the days preceding the study. People suffering from sleep, mental or neurological disorders were excluded. Ethical approval was granted by the committee of the Faculty of Medicine of the University of Liege (Belgium) and by the Ethical Committee of the Canton of Geneva (Switzerland).

#### 2.1.2 Patients with ND

Thirty‐six individuals (27 women and nine men) aged from 20 to 35 years old (M = 26 years, SD = 4.22) were included in the current analysis. Their characteristics are described in our previous publication (Schwartz et al., 2022). Participants were recruited at the Center for Sleep Medicine, University Hospitals of Geneva. Diagnosis of ND was done by a sleep specialist according to the International Classification of Sleep Disorders (ICSD‐3) diagnostic and coding manual (American Academy of Sleep Medicine, 2014). Signed informed consent was obtained from all participants before the experiment, and ethical approval for the study was obtained from the ethical committee of the canton of Geneva (Switzerland).

### 2.2 Experimental design

The experimental design of the study using IRT and TMR in the clinical population is described in detail in our previous paper (Schwartz et al., 2022). We will here provide a brief summary of the experiment. After the initial diagnosis and assessment of their ND (and depression scores), all participants filled in a dream diary at home for 2 weeks. At the end of this period, all patients had a single session of IRT, during which they were asked to change their initial nightmare in any way they wished, so that the new version would be neither unpleasant nor distressing. During a 5‐min period, half of the participants (IRT + TMR group, *N* = 18) received a 1‐s sound (i.e. the neutral piano chord C69, ∼42 dB) every 10 s while imagining the new dream scenario, while the other half did not receive the sound (IRT group, *N* = 18). During the following 2 weeks, all participants filled in a dream diary, they practiced imagery of the new scenario at home for 5 min per day (along with the sound for the IRT + TMR participants), and they also all received the sound every 10 s during REM sleep with a wearable headband electroencephalography sleep decoding device (Dreem; https://beacon.bio/dreem-headband/). At the end of this period, a second assessment session (post‐intervention) of the ND and depression scores took place. The 21‐item self‐rated Beck Depression Inventory (BDI‐II) was used for evaluating depressive symptoms (Beck et al., 1996) before and after the interventions.

### 2.3 Dream diaries

Participants in both groups (healthy participants, patients with nightmares) kept the same sleep and dream diary (for details, see Sterpenich et al., 2020). Every morning, upon spontaneous awakening, the participants were asked to report whether they had one or more dreams with or without recall or no dream at all just before awakening or occurring across the night. In case they remembered the content, they were also asked to freely write down the dreams they had experienced during their sleep.

In the healthy group, participants took part in different protocols in which they were requested to fill the diary for 1 week or for 3 weeks. The filling rate therefore varied across participants from 4 to 25 days, with a mean of 7.26 days, SD = 4.8. Although healthy participants may belong to different protocols, all of their dream diaries used in this study are “baseline”, with no experimental manipulation. The dream diary was given to participants of the clinical group for 14 days before IRT/TMR intervention and 14 days during the interventions.

No preprocessing was applied in the dream reports, and all analyses were performed on the raw data. In the healthy group, 475 dreams were collected, with a word average per dream = 63.5, SD = 64.5. In the clinical group, 313 dreams were collected before the interventions, with a word average per dream = 77.3, SD = 65.2, while 210 dreams were collected during the interventions, with a word average per dream = 64.8, SD = 54.6. There were no recalled written dreams for four participants in the dream diaries collected during the interventions.

Once each dream was classified under a specific category by the LLM algorithm (“nightmare”, “bad dream”, “cathartic dream”, “positive dream”, “neutral dream”; see later sections for more details), we calculated the proportion of dreams of each type and for each individual. To do so, the number of dreams of each type was divided by the total number of recalled dreams for the specific period. This resulted in one proportion for each of these five dream types for each subject. In the clinical group, there were two sets of values per patient, one for the 2‐week period before the interventions (e.g. Nightmare_pre, Bad_pre, Cathartic_pre, etc.) and one for the 2‐week period during the interventions (e.g. Nightmare_mid, Bad_mid, Cathartic_mid, etc.), and this, for participants in the IRT + TMR and IRT groups separately. We then took the average of these values across groups for descriptive statistics.

### 2.4 Criteria for dream content scoring for human scorers and the LLM algorithm

Both the AI algorithm and human scorers (whose scoring was used only for the validation of the algorithm) were given the primary definitions of the five types of dreams as shown in Table 1.

**TABLE 1:** Definitions of categories of dreams used for scoring from human scorers and the LLM algorithm.

Cathartic dream This is a dream that is characterized by the initial presence of danger, a difficult situation and negative emotions (e.g. fear, anxiety, anger, frustration), but which then evolves in the following way: Either a resolution of the problem: • The problem/danger diminishes or disappears on its own • The dreamer escapes or finds a solution • The danger is avoided Or a decrease in dream intensity after a climax: • Catastrophe is averted • The danger is still present but less intense Or a reduction in negative emotions: • I'm less afraid • I feel safer • I'm relieved, etc. Or the appearance of positive emotions (e.g. relief, joy, pride, a sense of justice, empathy). These are often dynamic dreams, with twists in the scenario.
Nightmare A dream with very intense negative emotions (e.g. fear, anger, frustration, anxiety) that often becomes increasingly disturbing and intense to a climax. Themes often include direct threats to survival, safety and physical integrity (e.g. violence, aggression, accidents, death, evil forces, abnormal/dangerous creatures). There is often (but not always) a night‐time awakening due to anxiety or negative emotion.
Bad dream A dream with little or moderately intense negative emotions, less than a nightmare. It usually does not wake the dreamer. Themes are more varied than nightmares, and these dreams often contain more interpersonal conflict than nightmares. Bad dreams are less bizarre (i.e. more rational and closer to everyday life) than nightmares.
Positive dream A dream with predominantly positive emotions (e.g. joy, tenderness, pride).
Neutral dream A dream with no emotions (positive or negative).

The definition of nightmares is inspired by the ICSD‐3 definition of nightmare (“*extended, extremely dysphoric, and well‐remembered dreams that usually involve threats to survival, security, or physical integrity*”; American Academy of Sleep Medicine, 2014), as well as the results of a large phenomenological study on nightmares and bad dreams (Robert & Zadra, 2014). The definition of bad dreams is also inspired by this study (Robert & Zadra, 2014). Nightmares were distinguished phenomenologically from bad dreams in this classification, as these two dreams seem to have different characteristics in terms of content and prevalence (Robert & Zadra, 2014). Previous studies that distinguished these two types (Robert & Zadra, 2014; Zadra & Donderi, 2000) were also based on the awakening criterion of DSM‐IV (nightmares = dreams with awakening; bad dreams = dreams without awakening). In the current classification, the awakening criterion (i.e. awakening due to anxiety or negative emotion) is not a necessary condition for nightmares, as it has been removed from the latest classifications of mental and sleep disorders (DSM‐V, ICSD‐3). To refine the categorization, supplementary and secondary input for both the human scorers and the algorithm was given as shown in Table 2. Examples of each of these five types of dreams are given in Supplementary Material S1.

**TABLE 2:** Supplementary and secondary rules for AI/human scoring.

Nightmare/Bad dream/Cathartic dream: If there's an awakening due to emotions, it's probably a nightmare If there's a resolution or reduction of the problem at the end, or any evolution “towards the better”, it's a cathartic dream If there's a reduction or lessening of negative emotions at the end, it's a cathartic dream If positive emotions appear at the end, it's a cathartic dream If negative emotions build to a climax, it's probably a nightmare If the negative emotions stagnate and evolve neither to a climax nor to something better as above, it's probably a bad dream If there's a threat to survival, safety and physical integrity, it's probably a nightmare If there are negative emotions with some form of violence, it's probably a nightmare
Interpreting emotions: Be careful not to invent emotions that are not present. For example, if it simply says “I was taking an exam”, you can't say there's fear, anxiety or concentration. On the other hand, if it says “I was stressed because of the exam”, you can say there's anxiety. The same goes for positive emotions; a wedding scene doesn't necessarily mean there's joy. If there are subjective judgements, you can consider them for emotions, e.g. “the exam was hard”, “the atmosphere was unpleasant”, “the people were nice”.
Dreams with several emotional changes: In cases where several emotional changes take place during a dream, the end of the dream counts most for its classification. In the example of one variation (negative‐>positive‐>negative), it could be classified as a bad dream or nightmare (depending on the respective criteria), while in the variation (positive‐>negative‐>positive), it would be a cathartic dream.
“Anti‐cathartic” dreams: We provisionally named as “anti‐cathartic” these dreams that start with a positive emotion and have a negative ending, and which should be scored as bad dreams or nightmares, according to the respective rules for these two types.

### 2.5 Description of the LLM algorithm

We used the gpt‐4o and gpt‐4o‐mini models from OpenAI (https://platform.openai.com/docs/models), prompted with the categories' definitions. To reduce the inherent non‐deterministic nature of LLMs, we used several agents and selected the classification with the most votes for each dream. The latest algorithm used gpt‐4o with nine agents.

The algorithm can be found in the following repository (https://github.com/oniri/cathartic-dreams), with step‐by‐step instructions to run the algorithm. The AI prompting is given in Supplementary Material S2. The algorithm reads dreams from an excel file, and concurrently sends the dream content to the OpenAI models, along with the instructions to classify the dream. It then computes the most voted classification among the nine agents and saves that classification. An OpenAI account (https://platform.openai.com/login) is necessary for its use.

### 2.6 Validation of the LLM

Before using the LLM algorithm in our dream datasets, we randomly chose 107 dreams collected from the studied populations, which were scored by three independent human scorers according to the described criteria, and were compared with classification from the LLM algorithm.

### 2.7 Statistical analysis

To validate the LLM algorithm against human classification, we calculated the kappa statistic as a measure of inter‐rater reliability between the scores of the human consensus (majority vote between the three human scorers) and those of the LLM algorithm (majority vote between the nine agents). To test the degree of non‐determinism of the algorithm (which is an inherent characteristic of LLMs), we performed five different trials of the algorithm on these 107 dreams and performed the Fleiss kappa test (inter‐rater reliability for >2 raters) for these five trials.

To study the effects of IRT and TMR on the outcome variables (proportion of cathartic dreams and negative dreams), data were entered into a multilevel regression model, with the outcome variables as dependent variables, and Time (pre‐, mid‐) and Group (IRT + TMR group, IRT group) as (interacting) independent variables. The latter represented the fixed effects of the multilevel model, while the random effects were represented by a random intercept for subjects. The random intercept accounted for correlation between repeated measures, by assuming baseline differences between subjects in the average dependent variable. A multilevel regression was chosen for these data, due to its ability to handle: (a) missing values; (b) time‐varying covariates; and (c) continuous within‐subject covariates.

Once the model was fitted, we performed a Type II ANOVA breakdown of fixed effects using *F*‐tests, starting with the interaction test of Time × Group, followed by main effects tests for Time and Group separately. Significant effects were further explored with pairwise *t*‐tests. Multiple testing correction for eventual follow‐up pairwise comparisons was done using the Holm–Sidak method (Holm, 1979). Degrees of freedom for all *F*‐ and *t*‐tests were adjusted for the random effects structure using Satterthwaite's method, yielding fractional degrees of freedom. Multilevel analyses were conducted with the R statistical language, version 1.2.5019 (RStudio Team, Boston, MA, USA, 2018; R Core Team, 2018), using the packages “lme4” for model estimation and “lmerTest” for inferential tests.

For the study of links between dream types and depression scores, the Kendall's tau correlation coefficient was calculated between the differences (subtraction) pre minus mid value for the five types of dreams (e.g. Cathartic_diff = Cathartic_pre – Cathartic_mid) and the difference pre minus post value for the BDI‐II depression scores (BDI_diff = BDI_pre – BDI_post).

## 3 RESULTS

### 3.1 Validation of the LLM algorithm against human classification

The agreement between the human consensus and the LLM algorithm for a pre‐selected 107 dreams was excellent (kappa = 0.844). There was 87% of agreement between the scorers and the algorithm for bad dreams, 82% for cathartic dreams, 89% for neutral dreams, 100% for nightmares, and 88% for positive dreams. The agreement between the five different LLM trials on these same 107 dreams was excellent (Fleiss kappa = 0.950).

### 3.2 Prevalence of emotional dreams in healthy and clinical populations

Overall, the dream recall frequency over all nights in the healthy population was 77%. In the nightmare group, the dream recall frequency was 70% before the interventions and 65% during the interventions. After its validation, the algorithm was used to score the larger dream databases (dreams of healthy participants: *n* = 475; and dreams of nightmare patients: *n* = 523). This analysis revealed that in healthy participants, 10% of dreams were scored as nightmares, 26% as bad dreams, 9% as cathartic dreams, 11% as positive dreams and 44% as neutral dreams (Table 3). On the other hand, in patients with ND, if both IRT and IRT + TMR groups are taken together before any intervention, the algorithm scored 37% of dreams as nightmares, 36% as bad dreams, 6% as cathartic dreams, 8% as positive dreams and 13% as neutral dreams. Table 3 also shows the prevalence of the five types of dreams when IRT and IRT + TMR groups were examined separately for both before and during the interventions. At pre‐intervention, no significant baseline differences were found between the two ND groups for the five types of dreams (*p* > 0.05).

**TABLE 3:** Prevalence of the five different types of dreams in healthy populations and patients with ND treated with IRT or combination of IRT and TMR.

	Healthy participants (*N* = 115)	IRT group (*N* = 18)		IRT + TMR group (*N* = 18)	
		Before intervention	During intervention	Before intervention	During intervention
Nightmares	10%	33%	17%	42%	16%
Bad dreams	26%	35%	43%	37%	23%
Cathartic dreams	9%	7%	11%	4%	25%
Positive dreams	11%	10%	11%	6%	18%
Neutral dreams	44%	15%	18%	11%	18%

Abbreviations: IRT, imagery rehearsal therapy; TMR, targeted memory reactivation.

### 3.3 Effect of IRT and IRT + TMR on proportion of cathartic dreams and negative dreams (i.e. nightmares and bad dreams)

**FIGURE 1:**
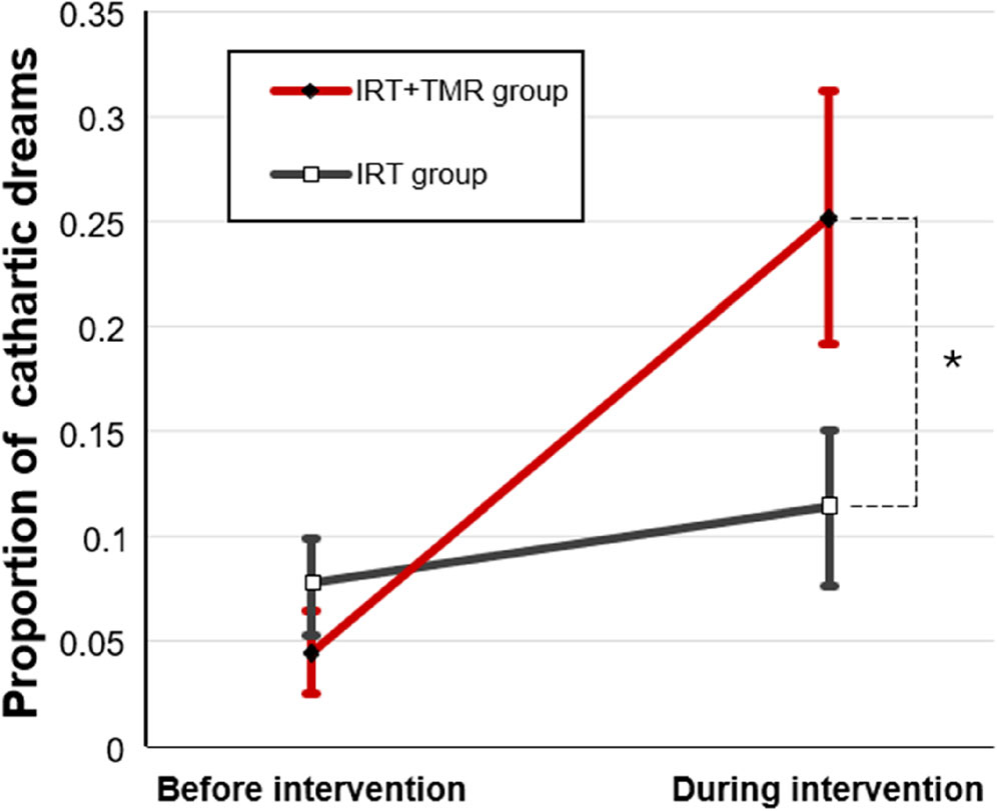
Treatment effects on the proportion of cathartic dreams in the two groups. Error bars represent standard errors of the mean (SEM). The asterisk (*) indicates statistically significant difference at the 0.05 level.

As expected, there was a significant time × group interaction (*p* = 0.02, *F*_1,34_ = 5.77), and a significant effect of time (*p* = 0.001, *F*_1,34_ = 12.44) for the percentage of cathartic dreams (Figure 1). A pairwise *t*‐test indicated that the IRT + TMR group had significantly more cathartic dreams than the IRT group during the intervention (*p* = 0.01, *t*_63.7_ = 2.60).

**FIGURE 2:**
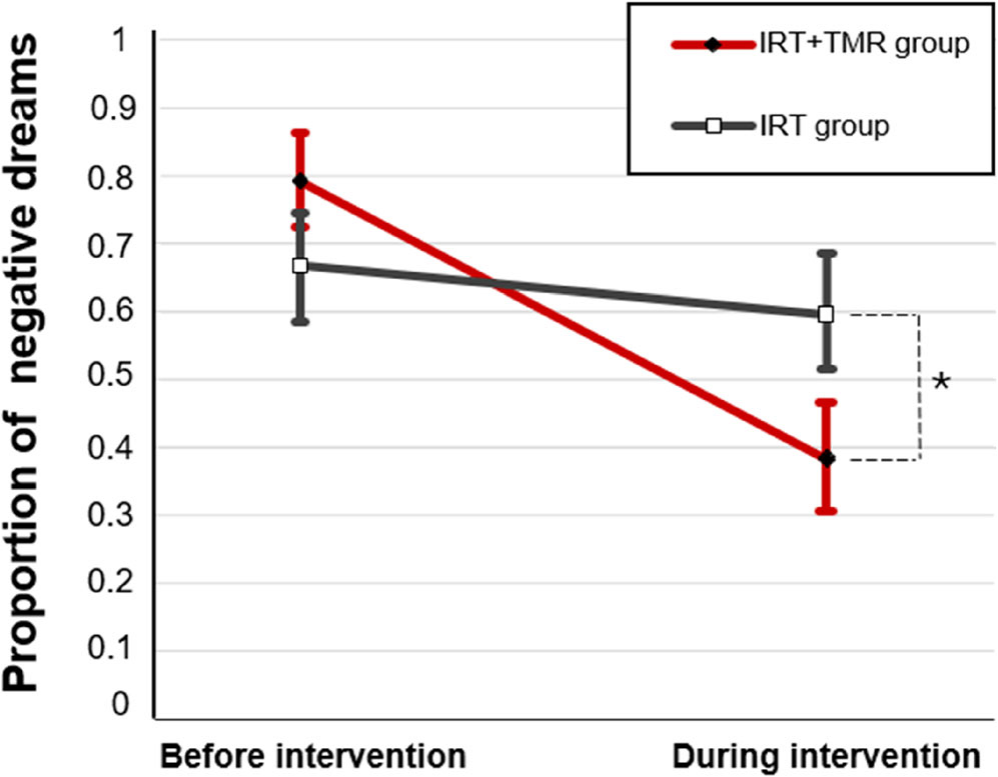
Treatment effects on the proportion of negative dreams (nightmares and bad dreams) in the two groups. Error bars represent standard errors of the mean (SEM). The asterisk (*) indicates statistically significant difference at the 0.05 level.

When nightmares and bad dreams were taken together (we here name both of them, negative dreams), there was a significant time × group interaction (*p* = 0.003, *F*_1,33_ = 10.07) and effect of time (*p* < 0.001, *F*_1,33_ = 20.33), while no group effect (*p* = 0.57, *F*_1.34_ = 0.31; Figure 2). A pairwise *t*‐test indicated that the IRT + TMR group had significantly less negative dreams than the IRT group during the intervention (*p* = 0.01, *t*_62.1_ = −2.46). Contrary to our hypothesis, there was no time × group interaction (*p* = 0.32, *F*_1,30_ = 1.02) or group effect (*p* = 0.48, *F*_1,31_ = 0.50) for the proportion of nightmares alone, while there was a significant effect of time (*p* < 0.001, *F*_1,30_ = 15.85). For the category of bad dreams, there was a significant time × group interaction (*p* = 0.03, *F*_1,33_ = 4.81) and no significant effect of time (*p* = 0.46, *F*_1,33_ = 0.5) or group (*p* = 0.15, *F*_1,33_ = 2.0). A pairwise *t*‐test indicated that the IRT + TMR group had significantly less bad dreams than the IRT group during the intervention (*p* = 0.01, *t*_63.7_ = −2.55). Moreover, the intervention had no significant effect on the proportion of positive dreams and neutral dreams (Supplementary Material S3).

### 3.4 Correlation of emotional dreams with depression scores

**FIGURE 3:**
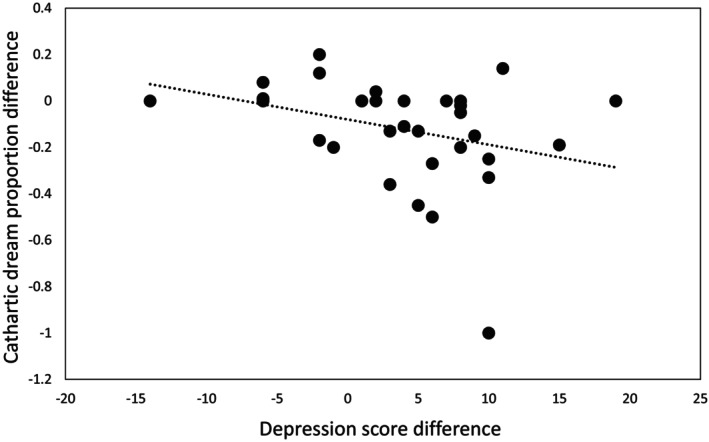
Correlation between cathartic dream proportion difference (Cathartic_pre – Cathartic_mid) and depression score difference (BDI_pre – BDI_post) in all patients with nightmare disorder (ND). Across participants, the Beck Depression Inventory (BDI) score difference was negatively correlated with the cathartic dream proportion difference (*p* = 0.008, τ = −0.313, one‐tailed). Each dot indicates a subject. The regression line is given for illustrative purposes.

In favour of our a priori hypothesis, cathartic dreams were the only type of dreams among the five types of dreams to correlate with depression scores. More specifically, among the differences between the scores of each of these five dream types before and during IRT therapy (Nightmare_diff, Bad_diff, Cathartic_diff, Positive_diff, Neutral_diff), only the Cathartic_diff was significantly correlated with BDI_diff (*p* = 0.008, τ = −0.313, one‐tailed; Figure 3), meaning that the more cathartic dreams increased in proportion during therapy in both groups, the more depression scores decreased. This correlation was significant for both the IRT group (*p* = 0.045, τ = −0.344) and the IRT + TMR group (*p* = 0.044, τ = −0.312), meaning this effect seems inherent to this type of dream during therapy and not to the type of interventions. No such correlation was observed for the other types (Nightmare_diff: *p* = 1, τ = 0.000; Bad_diff: *p* = 0.39, τ = 0.107; Positive_diff: *p* = 0.46, τ = −0.096; Neutral_diff: *p* = 0.64, τ = 0.059, two‐tailed).

## 4 DISCUSSION

The current study introduces and validates a new automatic algorithm based on LLM, which can be applied in large dream datasets in the future, following other similar promising endeavours (Bertolini et al., 2024; van Wyk et al., 2024). This not only allowed us to compute the prevalence of emotion in large sets of dream reports, but also to characterize a new type of dream, the cathartic one. This dream type seems to be independent of other emotional dreams, such as nightmares and bad dreams, as its prevalence increases during successful psychotherapy coupled with TMR. At the same time, it seems to have a function of “emotional thermostat”, as, among the five studied emotional types of dreams (nightmares, bad dreams, cathartic dreams, positive dreams, neutral dreams), it was the only one that correlated with depression scores in our clinical sample.

### 4.1 Prevalence of emotional valence in dreams of healthy and clinical populations according to the LLM algorithm

We first investigated the prevalence of emotions in 523 dreams of 115 healthy individuals by using a validated automatic external AI system applying the majority rule of nine different agents (LLM, OpenAI GPT). We have found that 44% of dreams were neutral, while 56% of dreams contained emotions. More specifically, 36% were negative, 11% were positive, while 9% were cathartic. These results are in accordance with other studies using external rating of emotional valence and finding a high percentage of neutral dreams (40%–70%) followed by negative dreams and then positive ones (Hall & Van de Castle, 1966; Schredl & Doll, 1998; Sikka et al., 2014). These external coding systems (which could be any external rater, including humans or AI, as in our case) usually code only emotions that are explicitly described in the dreams (this is also one of the rules that we gave to the algorithm; Table 2). On the other hand, subjective ratings of dream reports are usually more emotional than neutral (Nielsen et al., 1991; Sikka et al., 2014; St‐Onge et al., 2005), with a possible explanation being that participants can rate subtle moods that may not be transcribed in their dream reports and detected by external raters.

The prevalence of nightmares (10% of all dreams) and bad dreams (26% of all dreams) in our healthy population is higher than that found in a study with a larger sample in 2014 (*N* = 572, 10,000 dreams; 3% nightmares, 11% bad dreams; Robert & Zadra, 2014). This may be partly explained by the fact that the definitions given to the algorithm (and based on recent classifications of sleep disorders, ICSD‐3 and DSM‐5) may be more sensitive and that the awakening criterion is not necessary for scoring nightmares, as in the aforementioned study. Moreover, there may also be an increase in the prevalence of nightmares and bad dreams since the last 10 years, due for example to the COVID pandemic (Ableidinger et al., 2022).

To the best of our knowledge, it is also the first time to study the prevalence of emotional dreams using LLM in ND. We demonstrate that the emotional valence of dreams from this clinical population is, as expected, predominantly negative (73%), while 8% of dreams were positive, 6% cathartic and 13% of dreams neutral. We assume that, compared with the healthy participants, these patients, characterized by high rates of neuroticism, negativity bias and anxiety (Spoormaker et al., 2006; Zadra & Donderi, 2000), almost always describe explicitly their negative emotions in their dreams, which would also partly explain the decreased number of neutral dreams in this population, compared with the healthy one. Alternatively, neutral dreams (and potentially positive too, see later) could reflect a state of emotional stability, towards which psychotherapy and TMR gradually contribute. These assumptions need to be further investigated in the future.

### 4.2 Characteristics and functions of the cathartic dream

Cathartic dreams, which include switches from negative to positive emotions, appeared in a prevalence between 6% (ND—baseline) and 9% (healthy population). In the previous literature, this type of dreams may have been part of the so‐called “balanced dreams” (Röver & Schredl, 2017; Sikka et al., 2014), where both negative and positive emotions co‐exist within the same dream. In Robert and Zadra (Robert & Zadra, 2014), this type of dream was also included as a misidentified subcategory of the larger types of bad dreams and nightmares (“bad dreams and nightmares with positive ending”). However, we show that cathartic dreams follow an opposite direction in prevalence than nightmares and bad dreams during psychotherapy in our sample and, at the same time, they are the only dream type that negatively correlates with waking depression scores. This supports the idea that the resolution of negative emotions could be a key condition for a dream to have a function of emotion regulation. We thus propose that cathartic dreams should be considered as a separate and distinct type of dream.

In the past, several functions have been attributed to dreams, such as memory consolidation (Wamsley, 2014), creativity (Barrett, 2017), emotion regulation (Cartwright et al., 2006; Jung, 1969; Nielsen & Levin, 2007) or performance enhancement (Revonsuo, 2000). Other authors have also proposed that dreaming “serves” functions of sleep, such as brain maturation according to Hobson (Hobson, 2009) or synaptic homeostasis (Tononi et al., 2024). However, many psychoanalysts, psychologists and dream scientists seem to agree that a widely accepted function of dreams is that of emotion regulation (Perogamvros et al., 2013; Roesler, 2023; Zhang et al., 2024). Furthermore, the fact that the only type of dream that is considered pathological is one that strongly affects the emotions (nightmares), underlines that the emotional function of the dream is perhaps the most important among its potential functions.

Previous studies that explored the links between negative emotions in dreams and well‐being provided mixed results. Some studies supported that the presence of negative emotions in dreams may be sufficient for their emotion regulation function (Revonsuo, 2000; Sterpenich et al., 2020; Zhang et al., 2024), especially in good sleepers (Conte et al., 2021), while others have shown the opposite: that the more people experience negative emotions in their nighttime dreams, the more they express negative emotions and the less they report feeling positive affects the next morning (Mallett et al., 2022; Sikka et al., 2022), in accordance with the continuity hypothesis (Schredl & Hofmann, 2003). Importantly, the presence of disturbing dreams, such as nightmares, is associated with an increased risk of psychiatric and cardiovascular diseases (Nielsen & Carr, 2017). This observation confirms that any potential emotional learning in dreams fails when negative emotions are exaggerated and unresolved, as is the case in people suffering from ND.

One potential reason for the conflicting results of the aforementioned studies is methodological. The current analysis demonstrates that a dynamic and non‐binary analysis of the scenario and emotions in the dream may be more informative than a categorical (positive versus negative) analysis of dream content. Such an analysis should focus on the emotional switches/variations and problem‐solving within the same dream before concluding on a potential emotion regulation function of dreams.

Few studies have explored these aspects in dreams. In 2014, Robert and Zadra (Robert & Zadra, 2014) studied the content of bad dreams and nightmares, as well as the likelihood of a positive ending to these dreams, which means the transformation of a negative emotion into a positive one. In their study, approximately 20% of nightmares and 40% of bad dreams had a partially or completely positive ending. Nielsen and Carr later suggested that negative dreams with such positive resolution may be more functional in regulating emotions than those that lack resolution (Nielsen & Carr, 2017). Masset et al. (2023), by studying patients with REM sleep behaviour disorder episodes (i.e. acting out dreams in REM sleep), were able to further delve into the emotional dynamics of dreams. They found that a strong initial emotional increase (peaking at 10 min after the beginning in a REM behaviour episode), followed by a slow decrease during the remainder of the episode is observed. Likewise, negative emotions appeared earlier in REM sleep episodes than positive or neutral emotions. Moreover, valence shifts (negative to positive emotions or vice versa) were observed in approximately 31% of emotional REM dreams among participants in this study, further supporting an older study (Merritt, Stickgold, Pace‐Schott, & Williams, 1994).

We here propose that above and beyond the presence of negative emotions, what seems to render negative dreams potentially functional is their resolution. In that sense, not all dreams are functional. Indeed, the cathartic dreams, a specific type of dreams where negative emotions are replaced by positive ones, were the only type of dreams that negatively correlated with depression scores in our clinical population. More precisely, the higher the proportion of this type of dream increases during the interventions, the less depressed the participants were after the intervention compared with before. This correlation was not found for the other types of dreams. In a similar vein, in several empirical studies (Cartwright, 1991; Cartwright et al., 1998; Cartwright et al., 2006), Cartwright and colleagues have suggested that negative emotions in REM dreams need to be integrated with other autobiographical memories for the dream to have a beneficial emotion‐regulating function. Similarly, Hartmann (1996) proposed that dreaming allows us to link strong emotional memories to other related memories already stored in the brain to reduce the emotional intensity and distress caused by the experience (“calming the storm”). Nielsen and Levin (Nielsen & Levin, 2007) proposed that the main function of dreaming is to suppress conditioned fear, what we also call extinction. To achieve this function, frightening memories from the past (conditioned stimulus) are associated with new contexts, which are safe. The result of this process is that the conditioned stimulus gradually becomes neutral again, and no longer elicits the emotion of fear.

Our proposed functional classification of dreams does not grant the biggest importance to the intensity of negative emotionality of dreaming, but rather to the final resolution and relief of such negative emotions. As such, bad dreams are not necessarily more or less functional than nightmares, if we are based only on the degree of their emotional intensity. A final emotional resolution is conditional to the categorization of cathartic dreams. This resolution is reflected by closure (“complete act” according to Aristotle's definition) and relief, which distinguishes the cathartic dream from the (non‐cathartic) bad dreams and nightmares, both latter types being deprived of completion. This new experience would be internalized with the creation of a new “safety” memory in the hippocampus, which is consistent with Aristotle's idea that catharsis leads to learning and awareness of the individual, and therefore to pleasure.

### 4.3 The effect of IRT and TMR on dream content in patients with ND and comparison with our previous study

In our previous study (Schwartz et al., 2022) on the same ND participants undergoing 2‐week IRT and TMR interventions as described here, patients were asked to subjectively report the number of nightmares per week. In that study, we found that the IRT + TMR group had significantly less nightmares than the IRT group at the end of interventions. Interestingly, in the current objective analysis of the same data, a significant group difference is found only when nightmares and bad dreams are taken together. This can be explained by the fact that in the initial study, no specific distinction of nightmares and bad dreams was made. It may be then that some or all of our patients characterized most of their negative and disturbing dreams (including bad dreams) as nightmares.

Considering the results of the Schwartz et al. study and the current study together, we conclude that, while IRT alone can sufficiently reduce nightmares (Krakow et al., 2001), IRT + TMR can significantly reduce both nightmares and bad dreams, while also increasing cathartic dreams. These types of dreams seem interdependent in our study, in a way that the more negative dreams decrease, the more cathartic ones increase. This result supports previous studies showing how TMR in both non‐rapid eye movement (NREM; van der Heijden et al., 2024; Xia et al., 2024) and REM sleep (Greco et al., 2024; Hutchison et al., 2021; Rihm & Rasch, 2015; Schwartz et al., 2022) reduces the affective tone of previous aversive memories or it also increases their positive valence.

## 5 CONCLUSIONS AND LIMITATIONS

We have already argued that the use of LLM in our study, as any objective rating of dreaming, depends on explicit emotional reporting. Therefore, our study may have underscored the prevalence of emotional dreams, at least in the healthy population, as also shown in other studies comparing objective with subjective scoring of emotional valence (Schredl & Doll, 1998; Sikka et al., 2014). However, the prevalence of cathartic dreams may not have been much affected by this issue, as their criteria do not depend only on the emotional aspect, but also on effective problem‐solving and scenario twists (e.g. decrease of danger and increase of safety), which are usually adequately described in the dream texts.

Although preliminary, due to the rather small sample size of our clinical population, the results allow us to support the idea that TMR coupled with IRT can increase the proportion of cathartic dreams, which would ultimately promote a better emotional well‐being. Due to the sample and duration of the IRT in our study, we cannot exclude that IRT alone also increases both cathartic and positive dreams later on during therapy. In that sense, a previous study of traumatic nightmares (Germain et al., 2004) showed an increase in occurrences of friendliness, success and positive emotions, and a decrease in occurrences of aggression, failure and bad luck after a longer application of IRT. Positive dreams may thus reflect a state of mental balance in people who do not need to activate the cathartic function (so, at later stages of a successful psychotherapy). Longer psychotherapies, including different types (e.g. psychoanalysis, psychodynamic psychotherapy, cognitive‐behavioural therapy; Kempe et al., 2024), and bigger samples are needed to test this hypothesis in the future.

## AUTHOR CONTRIBUTIONS

**Lampros Perogamvros:** Conceptualization; funding acquisition; writing – original draft; writing – review and editing; visualization; supervision; formal analysis; methodology. **Vincent Rochas:** Formal analysis; writing – review and editing; visualization. **Jean‐Baptiste Beau:** Methodology; software. **Virginie Sterpenich:** Writing – review and editing. **Laurence Bayer:** Writing – review and editing; formal analysis; visualization.

## FUNDING INFORMATION

This study was supported by the Swiss National Science Foundation grants/award numbers CRSK‐3_190722 and 32003B_212588, and the Louis‐Jeantet Foundation (HUG Starter).

## CONFLICT OF INTEREST STATEMENT

The authors declare that they have no conflicts of interest related to this research.

## PATIENT CONSENTS PROTOCOL APPROVALS

Ethical approval was granted by the committee of the Faculty of Medicine of the University of Liege (Belgium) and by the Ethical Committee of the Canton of Geneva (Switzerland). All participants agreed to take part in this protocol and gave their written informed consent for the study. Registration number NCT05237778.

## Supporting information


**DATA S1** Supporting Information.

## Data Availability

The LLM algorithm can be found in the following repository (https://github.com/oniri/cathartic-dreams).
