# Supplementary material for: The cathartic dream: Using a large language model to study a new type of functional dream in healthy and clinical populations
Source: J Sleep Res. 2025 Feb 9;34(6):e70001. doi: 10.1111/jsr.70001 (PMC12592816; doi:10.1111/jsr.70001)
Supplement: Supplementary file 1 — DATA S1 Supporting Information. [file JSR-34-e70001-s001.docx]

**Supplementary Materials**

**S1. Examples of dreams**

Cathartic dream

*‘I was in a big house. A child was kidnapped and tied to a large double bed. He tried to wriggle free but could not. The child looked like my little brother, but the image was very blurred. I tried to save the boy at all costs, managed to get into the room he was trapped in and untied him, but when I got out, I came across a man who was making sure he didn't escape. Then he managed to get him back. At that moment, I felt quite angry. I then tried again to save the little boy and managed to get to an elevator where my friends were waiting. Just as I was about to get in, a woman tried to stop me from saving myself and the child. So I fought against her. Before the fight, this rebellious girl claims that she can beat me easily. I fight much better than her, I beat her. In the end, she's too exhausted and wounded to get back up. I feel a lot of pride.'*

Nightmare

*‘I was at my grandparent's country side house, having a large family reunion, sharing some food and drinks together. Suddenly, we started hearing noise of vehicles from afar, approaching towards the house. We looked through the window and could see these vehicles from the distance, since the house is located on the top of a small hill. We were very confused and scared. People finally reached the house and were wearing all black, a sort of ninja suit, we could see only their eyes. They placed bombs inside the house and they ran away from it. The bomb went off and there was a lot of fire and smoke. I was very angry, scared and confused, i wondered why did they attack us with no reason. I was desperately trying to find any of my other family members but couldn't find them.*

Bad dream

*‘I was at my dad's house at a family dinner. My brother decided to go outside to play and my father and stepmother started arguing. We sat around the table and I watched in embarrassment as the meal was getting cooked. Then my dad shows me his balcony, where he's growing an apple tree. Back at the table, they start arguing again. The pizza is still cooking, I can smell it, and my father and stepmother decide to divorce’.*

Positive dream

*‘I was with several of my friends in a bar and we had a big table. We had pitchers of beer and were drinking. I was so happy that the covid was over. We were all so happy. This bar was huge and had several floors. With a friend, we went upstairs and there was a room that was actually a soccer changing room. Inside, we found a World Cup trophy. We were surprised, but somehow we knew it belonged to Messi and that he was in the bar. I then went to another floor and there I bumped into a singer I love and we started mixing together. People were dancing and it was great. Then, with a friend of mine, we went to a church where there were objects like decorations, where we bumped into childhood friends and I saw that they were married and had children, and I said to myself that this is why they didn't go out with us’.*

Neutral dream

*‘I was dozing and could hear my mother on the phone. She was talking to my grandmother who wanted my mother to do her shopping. My mother didn't have the courage (it was very early in the morning) and told her she could give her some of the food we already had at home while my mother went to get my grandmother’.*

*‘I go to the market with some teenagers and buy some vegetables. I explain to a young man how to cut them. He asks me what's the hardest part about learning to cook. I tell him that at first, you prepare dishes where you don't really transform the food, and that's easy. It gets harder when cooking becomes abstract’.*

**S2. AI prompting**

You are a researcher studying dreams. Your role is to analyze dream reports and classify them into the following categories.

--------------------------

Classifications:

NIGHTMARE: nightmare

BAD: bad dream

CATH: cathartic dream

POS: positive dream

NEUTRAL: neutral dream

--------------------------

Complete Definitions:

Cathartic Dream

This is a dream characterized by the initial presence of danger, a difficult situation, and negative emotions (e.g., fear, anxiety, anger, frustration), but which subsequently evolves as follows:

a) Either a resolution of the problem:

The problem/danger diminishes or disappears on its own

The dreamer escapes or finds a solution

The danger is avoided

b) Or a decrease in the intensity of the dream after a climax:

The catastrophe is avoided

The danger is still present but less intense

c) Or a reduction of negative emotions:

"I'm less scared"

"I feel safer"

"I'm relieved"

etc.

d) Or the emergence of positive emotions (e.g., relief, joy, pride, a sense of justice, empathy)

These are often dynamic dreams, with twists in the storyline.

Nightmare

A dream with very intense negative emotions (e.g., fear, anger, frustration, anxiety) that often become more and more disturbing and intense until a climax. The themes often include direct threats to survival, safety, and physical integrity (e.g., violence, aggression, accidents, pursuit, death, evil forces, abnormal/dangerous creatures). There is often (but not always) nighttime awakening due to distress or negative emotions.

Bad Dream

A dream with low to moderately intense negative emotions, less intense than a nightmare. It usually does not wake the dreamer. The themes are more varied than nightmares and these dreams often include more interpersonal conflicts than nightmares. Bad dreams are less bizarre (i.e., more rational and closer to everyday life) than nightmares.

Positive Dream

A dream with primarily positive emotions (e.g., joy, tenderness, pride).

Neutral Dream

A dream with an absence of emotions (positive or negative).

--------------------------

Notes:

Nightmare / Bad Dream / Cathartic Dream:

If there is awakening due to emotions, it is probably a nightmare.

If there is resolution or reduction of the problem at the end, or any evolution "for the better," it is a cathartic dream.

If there is a reduction or attenuation of negative emotions at the end, it is a cathartic dream.

If there is the emergence of positive emotions at the end, it is a cathartic dream.

If there are negative emotions that build up to a climax, it is probably a nightmare.

If negative emotions stagnate and neither escalate to a climax nor improve as described above, it is probably a bad dream.

If there is a survival, safety, and physical integrity threat, it is probably a nightmare.

If there are negative emotions with some form of violence, it is probably a nightmare.

Interpretation of Emotions: Be careful, you should not invent emotions that are not present. For example, if it is simply mentioned, "I was taking an exam," you cannot say there is fear, anxiety, or concentration. However, if it is mentioned, "I was stressed about the exam," you can say there is anxiety. Similarly for positive emotions; a wedding scene does not necessarily mean there is joy. If there are subjective judgments, you can consider them for emotions, e.g., "the exam was hard," "the atmosphere was unpleasant," "the people were nice."

Dreams with Multiple Emotional Changes: In cases where multiple emotional variations occur during a dream, the end of the dream matters most for its classification. For example, in a variation (negative -> positive -> negative), it could be classified as a bad dream or nightmare (depending on the respective criteria), whereas in the variation (positive -> negative -> positive), it would likely be a cathartic dream.

‘Anti-Cathartic’ Dreams: Dreams that start with a positive emotion and end negatively should be scored as bad dreams or nightmares.

------------------------------

Here is the dream:

[DREAM]

------------------------------

What is your classification? Return the following JSON format:

{ 'reason': reason for the classification, max 2 sentences, answering the "if there is" questions above in order of relevance and stopping as soon as the answer is clear, 'classification': classification }

JSON:

**S3. Effects of interventions on the proportion of positive dreams and neutral dreams**

For the category of positive dreams, there was no time*group interaction (p = 0.20, F(1,64) = 1.62) or group effect (p = 0.80, F(1,64) =0.05) or time effect (p = 0.14, F(1,64) = 2.23).

For the category of neutral dreams, there was no time*group interaction (p = 0.46, F(1,32) = 0.55) or group effect (p = 0.62, F(1,34) =0.24) or time effect (p = 0.08, F(1,32) = 3.16).
